# Straw Soil Conditioner Modulates Key Soil Microbes and Nutrient Dynamics across Different Maize Developmental Stages

**DOI:** 10.3390/microorganisms12020295

**Published:** 2024-01-30

**Authors:** Jianfeng Zhang, Libo Ye, Jingjing Chang, Enze Wang, Changji Wang, Hengfei Zhang, Yingnan Pang, Chunjie Tian

**Affiliations:** 1 College of Life Science, Jilin Agricultural University, Changchun 130118, China; zhangjianfeng06@tsinghua.org.cn (J.Z.); yelibo@iga.ac.cn (L.Y.); zhanghengfei059868@163.com (H.Z.); 13943170420@163.com (Y.P.); 2 Northeast Institute of Geography and Agroecology, Chinese Academy of Sciences, Changchun 130102, China; changjingjing@iga.ac.cn (J.C.); wangenze@iga.ac.cn (E.W.); wangchangji@iga.ac.cn (C.W.)

**Keywords:** soil amendments, straw-based carbon substrate, microbial communities, soil nutrients, crop yield

## Abstract

Soil amendments may enhance crop yield and quality by increasing soil nutrient levels and improving nutrient absorption efficiency, potentially through beneficial microbial interactions. In this work, the effects of amending soil with straw-based carbon substrate (SCS), a novel biochar material, on soil nutrients, soil microbial communities, and maize yield were compared with those of soil amendment with conventional straw. The diversity and abundance of soil bacterial and fungal communities were significantly influenced by both the maize growth period and the treatment used. Regression analysis of microbial community variation indicated that Rhizobiales, Saccharimonadales, and Eurotiales were the bacterial and fungal taxa that exhibited a positive response to SCS amendment during the growth stages of maize. Members of these taxa break down organic matter to release nutrients that promote plant growth and yield. In the seedling and vegetative stages of maize growth, the abundance of Rhizobiales is positively correlated with the total nitrogen (TN) content in the soil. During the tasseling and physiological maturity stages of corn, the abundance of Saccharimonadales and Eurotiales is positively correlated with the content of total carbon (TC), total phosphorus (TP), and available phosphorus (AP) in the soil. The results suggest that specific beneficial microorganisms are recruited at different stages of maize growth to supply the nutrients required at each stage. This targeted recruitment strategy optimizes the availability of nutrients to plants and ultimately leads to higher yields. The identification of these key beneficial microorganisms may provide a theoretical basis for the targeted improvement of crop yield and soil quality. This study demonstrates that SCS amendment enhances soil nutrient content and crop yield compared with conventional straw incorporation and sheds light on the response of soil microorganisms to SCS amendment, providing valuable insights for the future implementation of this material.

## 1. Introduction

A common strategy for ameliorating soil degradation is returning straw to the soil to improve soil aggregation [1,2], structure, aeration, water holding capacity, and cation exchange capacity [3,4,5,6,7]. However, the high carbon/nitrogen (C/N) ratio of straw and the presence of polymers such as cellulose and lignin hinder its degradation. In regions with cold temperatures, such as northern China, high latitudes and climate change further reduce straw degradation [8,9,10]. Undecomposed straw can affect crop germination and root growth, as well as subsequent crop yield and quality [11], and provides a favorable environment for pathogen proliferation and pest egg survival. This can significantly increase the risk posed by crop pests and diseases, such as corn root rot, wheat sharp eye disease, and corn borer infestation [12,13,14]. These limitations of traditional straw management practices underscore the urgent need for alternative strategies to mitigate soil degradation.

One effective option is the amendment of the soil with soil conditioners produced from straw. Typically, high-temperature pyrolysis is used to produce carbonaceous products from straw, which are then used as soil conditioners [15]. This study uses a novel method for processing straw in which crushed straw is treated under hydrothermal conditions at high temperature and pressure to produce a straw carbon matrix material called straw-based carbon substrate (SCS). Following air-drying, SCS retains the abundant nutrients present in straw while minimizing the risks associated with insect eggs and disease. This method also reduces carbon loss [16].

Compared with traditional straw return methods, processing straw into a soil amendment significantly improves soil nutrient availability and crop yields [17,18]. In short-term field trials, the addition of soil amendments greatly increased soil enzyme activity and soil microbial populations [15,19]. Soil amendments can shape microbial network patterns, with varying effects across different tillage layers [20]. These amendments may stimulate the growth of beneficial soil microorganisms that enhance plant growth through nutrient release and hormone production. However, the effects of soil amendments on soil chemical properties and microbial communities have not been examined during the different stages of plant growth. This study addresses this gap by assessing the effects of SCS on microbial communities throughout maize growth using high-throughput sequencing. The objectives were to explore and characterize potential microbial soil amendments and to identify key beneficial microorganisms at different growth stages to provide a theoretical basis for targeted improvements in crop yield and soil quality.

## 2. Materials and Methods

### 2.1. Site Description

The experiments in this study were carried out in Dongfeng County, Liaoyuan City, Jilin Province, China (125°43′ E, 42°54′ N), which is located in a semi-humid continental climate zone with a temperate climate. In this region, the average annual temperature is 5.2 °C, and the average annual precipitation is 658.1 mm. Rainfall primarily occurs from May to August, with the highest amount of rainfall occurring in July. The soil in this area is classified as black soil.

### 2.2. Experimental Design and Soil Sampling

There were two sets of treatments in this experiment, each of which was replicated four times, and all treatments were 10 × 10 m in size, one for each treatment. In the common stover (CK) treatment, 100 kg of locally produced maize stover crushed to a size of approximately 5 cm was spread evenly over the soil surface. In the straw carbon matrix (CM) treatment, 100 kg of SCS was spread evenly over the soil surface.

To prepare SCS, corn stalks were crushed into 2–3 cm pieces, mixed with water, and heated at 160 °C for 2 h. Next, butanol was added at a ratio of 1:1 by volume, and the material was heated at 200 °C for 2 h. The material was then cooled and centrifuged to obtain a solid product with a water content of 10–40%. After drying at room temperature, the SCS was obtained. Under the hydrothermal processing conditions, the cellulose and hemicellulose in the straw were hydrolyzed and decomposed into soluble organic molecules, ultimately changing the structure and composition of the straw. 

Maize was planted on 11 May 2022 and harvested on 8 October 2022. Soil samples were collected on the fourteenth day of each month from the seedling stage to physiological maturity. The soil samples were collected using a 20 cm long auger with a diameter of 38 mm. Five soil samples were collected from the plots of each treatment group, mixed evenly, bagged, and stored at 4 °C for soil analysis or −80 °C for microbial DNA extraction.

### 2.3. Soil Physicochemical Analysis

Soil pH and electrical conductivity (EC) were measured at a soil:water ratio of 1:2.5 (*w*/*v*) with the use of a pH meter (Metro-pH 320; Mettler-Toledo Instruments Ltd., Shanghai, China) [21]. Total nitrogen (TN) and total carbon (TC) were determined with the use of a 2400 Series II CHNS/O System elemental analyzer (PerkinElmer Instruments Ltd., Shanghai, China). Total phosphorus (TP) was determined using inductively coupled plasma–atomic emission spectrometry (ICP-AES; Profile DV, Teledyne Leeman Labs, Hudson, NH, USA) after reflux digestion with hydrofluoric acid, nitric acid, and perchloric acid. Soil-available phosphorous (AP) was extracted with 0.5 M NaHCO_3_ and determined with the molybdenum antimony colorimetric method [22].

### 2.4. DNA Extraction and High-Throughput Sequencing

Soil microbial DNA was extracted from 0.5 g of soil with the use of a Quick-DNA SPIN Kit (MP Bio Laboratories, Carlsbad, CA, USA) according to the manufacturer’s instructions. The quality and quantity of the DNA were evaluated using a NanoDrop 2000 spectrophotometer (Thermo Scientific, Schwerte, Germany) after the DNA was dissolved in sterile distilled water. Bacterial community composition was determined by amplifying the V3-V4 region of the bacterial 16S rRNA gene with the use of the primer sequences 341F 5′-ACTCCTACGGGAGGCAGCA-3′ and 785R 5′-GGACTACHVGGGTWTCTAAT-3′ [23]. Fungal community composition was determined by amplifying the ITS1 region using the primer sequences ITS1F 5’-CTTGGTCATTTAGGAAGTAA3′ and ITS2R 5′-GCTGCGTTCTTCATCGATGC-3′ [24,25,26]. The amplicons were used to construct libraries, which were sequenced on the Illumina NovaSeq platform (Illumina, San Diego, CA, USA). The raw sequencing data were analyzed using QIIME2 (https://view.qiime2.org/)).

Paired-end sequences from the bacterial data were merged using the vsearch plugin and quality filtered. The resulting valid sequences were processed using the deblur plugin to remove redundant sequences and obtain representative sequences. The fungal data were denoised using the data2 plugin to obtain representative sequences and a redundant sequence feature table. The phylogeny plugin was used to construct a phylogenetic tree from representative sequences. The diversity plugin was used to calculate core diversity metrics, such as the Shannon index, Bray–Curtis clustering, and UniFrac distance matrices. Bacterial taxonomy was based on the Greengenes database gg_13_8 with 99% similarity, and fungal taxonomy was based on the UNITE database version 7.1 using the feature-classifier plugin with 97% sequence similarity. Finally, representative operational taxonomic units (OTUs) were identified with the use of Usearch v.10 [27].

### 2.5. Statistical Analyses

We performed a one-way analysis of variance (ANOVA) and carried out Duncan’s test with the use of SPSS software (version 19.0) to analyze differences in soil properties. Statistical analyses were performed using R Studio version 1.3.959 with R version 4.02 [28]. The generalized joint attribute modeling (GJAM) package was used to estimate the effects of the SCS soil amendment treatments [29]. The regression coefficients for each treatment were extracted from the model to identify treatment-induced shifts in the abundance of microbial communities (bacterial and fungal). After 10,000 simulations, the stability of the estimated coefficients was confirmed using Markov chain Monte Carlo (MCMC) evaluation. These coefficients were then used during principal coordinate analysis (PCoA) to explore the similarities between the communities in the different treatments and to highlight shifts induced by the treatments. The significance of clustering was analyzed using the hierarchical clustering on principal components (HCPC) procedure in the FactoMineR package [30]. In addition to the intensity of the shifts in the relative abundance of the soil microbiome provided by the GJAM regression coefficients, the relevance of the different microbes for the soil community was evaluated in terms of the centered log-ratio (CLR) transformed abundance [31]. CLR transformation gives the importance of different microbial groups (bacterial or fungal) as a proportion of the sample average. This transformation was used to classify the soil microbes as being in originally high or low abundance based on log-fold differences relative to the mean, which is zero in CLR-transformed data.

## 3. Results

### 3.1. Soil Properties

As shown in Table 1, soil TC and TN were significantly higher (*p* < 0.05) in the CM treatment than in the CK treatment in all four maize growth stages: seedling, vegetative, silking, and physiological maturity. At maize physiological maturity, the soil pH was acidic, and soil salinity (as measured with the content of EC) was significantly higher in the CM treatment than in the CK treatment. However, soil TC, TN, TP, and AP decreased in the CM treatment in the silking stage compared with the vegetative stage. Overall, the contents of soil nutrients were higher in the CM treatment than in the CK treatment at all maize growth stages.

### 3.2. Microbial Beta Diversity

**Figure 1:**
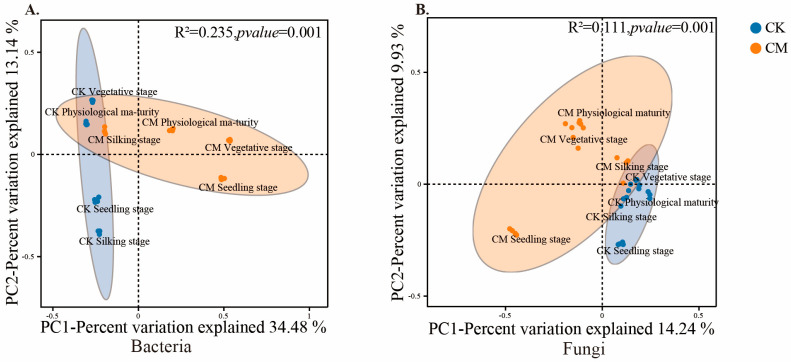
Principal coordinate analysis (PCoA) plot of changes in soil bacterial and fungal communities during the different growth stages of maize treated with CK and SCS (CM). PCoA plots for soil (**A**) bacterial and (**B**) fungal communities at the maize seedling, vegetative, silking, and physiological maturity stages. Distances between the samples, based on similarity in OTU composition (OTU similarity ≥ 97%) calculated with Spearman_approx distance, are visualized by principal coordinates analysis (PCoA) plots. A greater distance between two samples infers a lower level of similarity. The percentage of variation explained by PC1 and PC2 is indicated in the axis.

Differences in the soil bacterial and fungal communities between the treatments and among the maize growth stages were analyzed with the construction of PCoA plots at the OTU level, which are shown in Figure 1. The bacterial and fungal communities differed between the treatments in all maize growth stages. The variation in bacterial community explained by the first two axes of the PCoA plot was 47.62% (Figure 1A, R^2^ = 0.235, *p* < 0.05). The variation in fungal community explained by the first two axes of the PCoA plot was 24.17% (Figure 1B, R^2^ = 0.111, *p* < 0.05).

### 3.3. Microbial α-Diversity

**Figure 2:**
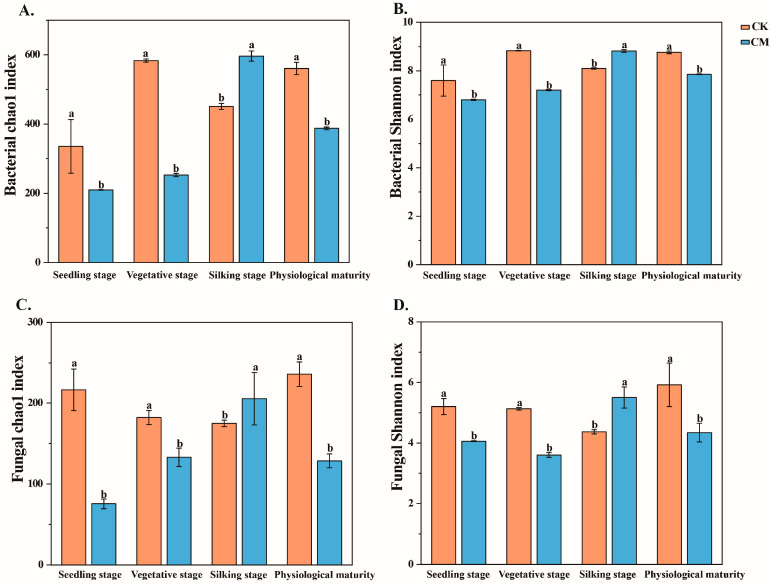
Chao1 and Shannon indices of bacterial and fungal communities under two treatments (CK and CM) at the maize seedling stage, vegetative stage, silking stage, and physiological maturity. The values shown are the mean ± SD (standard deviation). Data with the same letters within each column indicate no significant difference using one-way ANOVA with LSD tests at *p* < 0.05. The Chao1 index of bacteria (**A**), Shannon index of bacteria (**B**), Chao1 index of fungi (**C**), and Shannon index of fungi (**D**) for soil microbes during the maize seedling stage, vegetative stage, silking stage, and physiological maturity.

The diversity (Shannon) and richness (Chao1) indices of the soil bacterial and fungal communities were significantly lower in the CM treatment than in the CK treatment at the maize seedling stage, vegetative stage, and physiological maturity (Figure 2). These indices were also significantly higher in the CM treatment than in the CK treatment at the maize silking stage.

### 3.4. Relationships between Regression Coefficients and Bacterial and Fungal Abundance

**Figure 3:**
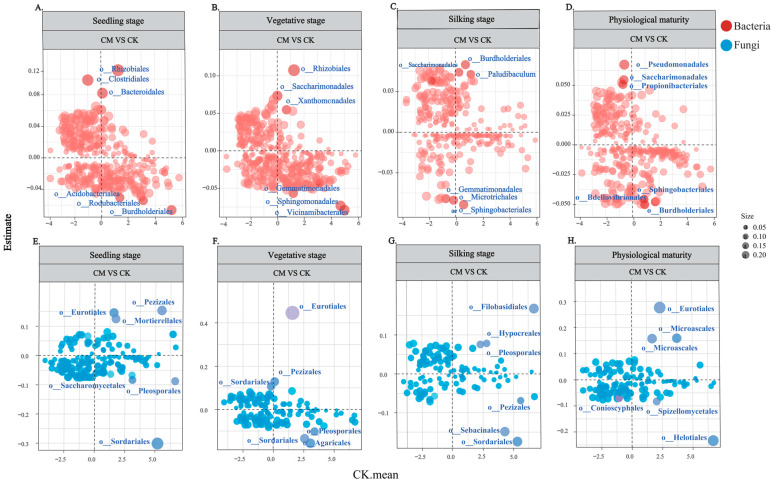
Relationships between regression coefficients and bacterial and fungal abundance (CLR-transformed). The three most significant negative and positive changes in bacterial and fungal taxa in the CM treatment compared with the control treatment at the maize seedling, vegetative, silking, and physiological maturity stages were determined. “Unc.” refers to unclassified taxa. A coefficient was considered significant when its confidence interval was 95%. The most significant negative and positive changes in bacterial taxa during the seedling stage are denoted as (**A**), during the vegetative stage as (**B**), during the silking stage as (**C**), and during the physiological maturity stage as (**D**). The most significant negative and positive changes in fungal taxa during the seedling stage are denoted as (**E**), during the vegetative stage as (**F**), during the silking stage as (**G**), and during the physiological maturity stage as (**H**). Size value, the larger the "a" circle, the greater the impact of this treatment. The darker colors indicate the three groups that are most positively and negatively correlated with processing.

The relationships between regression coefficients and microbial community abundance (CLR-transformed) were analyzed to identify the top bacterial and fungal taxa that were positively and negatively correlated with the CK treatment (Figure 3). At the seedling stage, when nodulation occurs, the CM treatment was positively correlated with changes in the bacterial taxon abundance of Rhizobiales, Clostridiales, and Bacteroidales and negatively correlated with Acidobacteriales, Rodubacteriales, and Burkholderiales. At the vegetative (mating) stage, the CM treatment was positively correlated with Rhizobiales, Saccharimonadales, and Xanthomonadales and negatively correlated with Gemmatimonadales, Sphingomonadales, and Vicinamibacterales. At the silking (filling) stage, the CM treatment was positively correlated with Burdholderiales, Saccharimonadales, and Paludibaculum and negatively correlated with Gemmatimonadales, Microtrichales, and Sphingobacteriales. At physiological maturity, the CM treatment was positively correlated with Pseudomonadales, Saccharimonadales, and Propionibacteriales and negatively correlated with Sphingobacteriales, Bdellovibrionales, and Burdholderiales. Interestingly, Rhizobiales was consistently highly positively correlated with the CM treatment at the seedling and vegetative stages, whereas Saccharimonadales was positively correlated with the CM treatment during the vegetative, silking, and physiological maturity stages. These results demonstrate that SCS application affects specific bacterial taxa that may hold potential for development as targeted microbial interventions in agricultural practices.

In the fungal community, the CM treatment was positively correlated with Pezizales, Eurotiales, and Mortierellales and negatively correlated with Saccharomycetales, Pleosporales, and Sordariales at the seedling (nodulation) stage. At the vegetative stage, the CM treatment was positively correlated with Eurotiales, Pezizales, and Sordariales and negatively correlated with Agaricales, Sordariales.1, and Pleosporales. At the silking (filling) stage, the CM treatment was positively correlated with Filobasidiales, Hypocreales, and Pleosporales and negatively correlated with Sebacinales, Sordariales, and Pezizales. At physiological maturity, the CM treatment was positively correlated with Eurotiales, Microascales, and Microascales.1 and negatively correlated with Conioscyphales, Spizellomycetales, and Helotiales. The strong positive correlation between Eurotiales and the CM treatment in the seedling, vegetative, and physiological maturity stages suggests that this fungal taxon is a key player in the fungal community’s response to SCS application.

### 3.5. Correlation between Soil Microbial Community and Soil Physiochemical Properties

**Figure 4:**
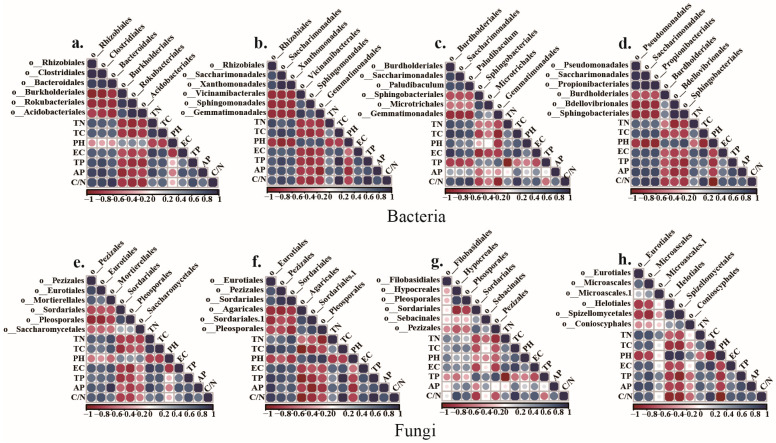
Correlation between the abundance of core bacterial (**a**–**d**) and fungal taxa (**e**–**h**) and soil chemical parameters. The values were determined at the different maize growth stages: seedling (**a**,**e**), vegetative (**b**,**f**), silking (**c**,**g**), and physiological maturity (**d**,**h**). Positive correlations are shown in blue, and negative correlations are shown in red. The color of the dots represents the proximity of the correlation coefficient to 1 or −1, with darker blue dots indicating coefficients closer to 1 and darker red dots indicating coefficients closer to −1. The size of the dot indicates the magnitude of the correlation coefficient between the two variables.

To estimate the correlations between the abundance of key bacterial and fungal taxa and soil physicochemical properties at all stages of maize growth, we used the Spearman rank correlation to generate correlation heatmaps (Figure 4). During the seedling (nodulation) stage (Figure 4a), we observed that the abundance of key core bacterial taxa Rhizobiales, Clostridiales, and Bacteroidales in the CM treatment was strongly correlated with soil TN (total nitrogen), TC (total carbon), EC (electrical conductivity), TP (total phosphorus), AP (available phosphorus), and the C/N ratio. These results indicate that the taxa significantly affect these soil physicochemical properties. The bacterial taxa Burkholderiales, Acidobacteriales, and Rokubacteriales, which were negatively correlated with the CM treatment, were strongly correlated with pH.

At the vegetative (male withdrawal) stage, the bacterial taxa Rhizobiales, Saccharimonadales, and Xanthomonadales, which were positively correlated with the CM treatment, were strongly correlated with soil TN, TC, EC, TP, AP, and the C/N ratio (Figure 4b). In the seedling and vegetative stages, soil TN, TC, EC, TP, AP, and the C/N ratio were higher in the CM treatment than in the CK treatment (Table 1), suggesting that straw was more readily decomposed in the CM treatment. Among these taxa, the abundance of Rhizobiales was highest.

During the silking (grubbing) stage, the bacterial taxa Burdholderiales, Saccharimonadales, and Paludibaculum, which were positively correlated with the CM treatment, were strongly correlated with soil TN, TC, EC, TP, AP, and the C/N ratio (Figure 4c). As shown in Table 1, soil TN, TC, EC, TP, AP, and the C/N ratio started to decrease in the silking stage.

At physiological maturity, the bacterial taxa Pseudomonadales, Saccharimonadales, and Propionibacteriales, which were positively associated with the CM treatment, were strongly correlated with soil TN, TC, EC, TP, AP, and the C/N ratio (Figure 4d). The abundances of Saccharimonadales and Xanthomonadales were also relatively high during the vegetative stage.

At the seedling and vegetative stages, the core fungal taxa that were positively correlated with the CM treatment, namely, Pezizales, Eurotiales, Mortierellales, and Sordariales, were strongly correlated with soil TN, TC, EC, TP, AP, and the C/N ratio (Figure 4e,f). Members of the Eurotiales, Mortierellales, and Sordariales families are known fungal decomposers with the ability to break down complex organic materials, including cellulose, hemicellulose, proteins, and lipids, into simpler organic compounds. In contrast, at the silking and physiological maturity stages, soil TN, TC, EC, TP, AP, and the C/N ratio were strongly positively correlated with Filobasidiales and Eurotiales and positively correlated with Microascales, Hypocreales, and Pleosporales (Figure 4g,h). Similar core taxa were positively correlated with the CM treatment in these two maize stages.

### 3.6. Crop Yield

**Figure 5:**
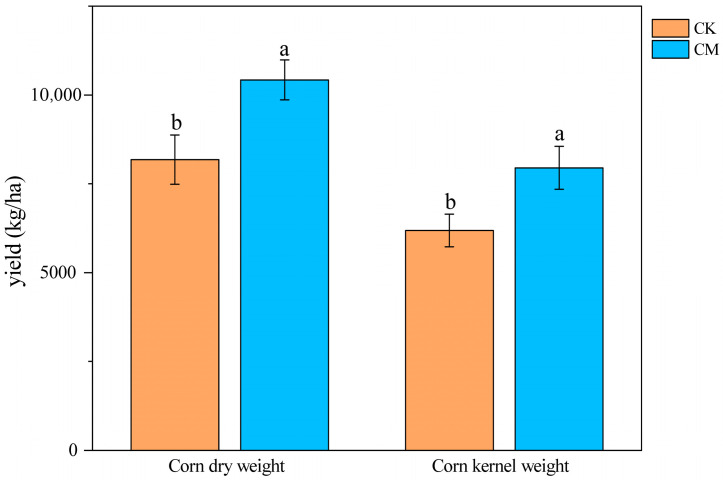
Dry weight and kernel weight yield of maize per hectare in the CK and CM treatments. Different lowercase letters for a parameter indicate significant differences between the treatments at *p* = 0.05.

Compared with the CK treatment, the grain dry weight and kernel weight of maize per hectare were 12.06% and 12.48% higher, respectively, in the CM treatment; thus, both differences were significant (Figure 5).

## 4. Discussion

Soil amendment is a promising strategy for improving soil quality. Soil amendment has multiple benefits, including increasing soil organic carbon (SOC) levels [32], water-holding capacity [3,33], aeration, basal saturation, and nutrient retention and availability; reducing fertilizer requirements and nutrient leaching; stimulating microbial biomass and activity; improving crop growth and yields; and increasing carbon sequestration [4,5,34]. The sequestration of carbon in soil also contributes to overall soil quality and the sustainable utilization of natural resources. In a study of saline soil in China, Lashari et al. found that applying soil amendments and kerosene solution significantly improved soil properties and maize plant performance and yields; these beneficial effects increased over time [18]. In Laos, applying soil amendments to low-fertility soils improved soil quality, plant performance, and maize yields [35]. However, the changes in soil dynamics and microbial communities during the different stages of crop growth have not been comprehensively examined. This study investigated these changes to gain a deeper understanding of the mechanisms underlying the effects of soil amendments on soil quality and crop productivity.

The soil microbial community structure was influenced by the maize growth period and the soil amendment treatment. PCoA revealed significant differences in the temporal variations in the soil microbial communities between the two treatment groups, and α-diversity analysis demonstrated that the richness and diversity of the soil microbial communities were significantly influenced by the treatments. The soil microbial community is influenced by the different plant growth stages due to changes in the substances secreted into the rhizosphere by the plant [36,37,38]. The decomposition of straw and SCS causes the release of nutrients into the soil. Depending on the rate of decomposition, nutrient release can stimulate different levels of soil microbial diversity and richness [39,40]. In this study, the diversity and abundance of soil bacteria and fungi were lower in the CM treatment than in the CK treatment in the seedling, vegetative, and physiological maturity stages. Rapid plant growth increases competition for nutrients, reducing microbial growth. As the organic matter in straw and SCS was gradually utilized and decomposed by microorganisms, microbial abundance and diversity increased, reaching a peak in the silking stage and declining with decreasing temperatures at physiological maturity. 

The relationships between regression coefficients and abundance were analyzed to identify the fungal and bacterial taxa that were most significantly affected by the CM treatments in each maize growth stage. Rhizobiales showed the most significant variations at the seedling and vegetative stages. Members of this bacterial family, such as *Rhizobium*, form symbiotic relationships with plant roots [41]. *Rhizobium* forms mycorrhizal symbioses with the rhizomes of legumes [42]. During the seedling and early growth stages, rhizobia sense chemical signals released by the plant’s root system and establish symbiotic relationships by infecting root hair cells [43]. In non-leguminous higher plants, such as sugarcane and *Oryza sativa*, rhizobia fix nitrogen [44,45]. We hypothesize that during the initial stages of maize growth, specific interactions between *Rhizobium* and maize occur that lead to increases in the relative abundance of *Rhizobium* and nitrogen sources, thereby positively influencing plant growth and development [46]. Clostridiales are capable of breaking down organic matter and releasing nutrients, such as nitrogen, phosphorus, and potassium, and micronutrients for plant uptake, promoting plant growth [47,48]. The abundance of Saccharimonadales increased in the maize vegetative, silking, and physiological maturity stages. These bacteria may be associated with sugar breakdown and utilization [49,50] and are potential candidates for promoting plant growth.

The three fungal taxa that were most positively affected by the CM treatment in the maize growth stages were Pezizales, Eurotiales, and Filobasidiales. Pezizales and Eurotiales belong to the phylum Ascomycota and play important roles in the carbon and nitrogen cycles. These fungi break down complex organic matter, such as plant debris and humus, into smaller organic molecules [51], which facilitates the cycling of organic carbon and its release to other organisms. In addition, Pezizales and Eurotiales form mycorrhizal symbioses with plants that increase organic nitrogen uptake from the soil and promote nitrogen cycling and utilization. Filobasidiales, in synergy with other microorganisms, can decompose more easily degradable components in straw, such as certain sugars or proteins. Some Filobasidiales form mycelial networks that improve soil structure and increase aeration and water retention [52], positively affecting soil health and plant growth. Overall, these three fungal taxa are involved in different aspects of carbon and nitrogen cycling in the soil, including organic matter decomposition and degradation, mycorrhizal symbiosis, and soil structure improvement. Their activities are important for the maintenance of soil quality and ecosystem functioning. Further research on the ecological roles of these fungi may contribute to better the management of soil resources, ecological balance, sustainable agriculture, and ecosystem health.

In both treatment groups, soil carbon, nitrogen, and phosphorus were significantly correlated with the abundance of specific soil microorganisms [53,54]. This finding suggests that soil microorganisms play a crucial role in the transformation of incorporated straw into soil nutrients. Notably, Saccharimonadales abundance was significantly positively correlated with soil TC, TN, TP, and AP at all stages of maize growth. Some members of Saccharimonadales produce alkaline phosphatase, an enzyme that is involved in the decomposition and transformation of organic phosphorus and facilitates phosphorus and nutrient cycling [55].

## 5. Conclusions

In this study, we investigated the effects of soil amendment with SCS on soil nutrient content and the soil microbial community. At the seedling and vegetative stages of maize, the bacterial and fungal taxa most strongly associated with SCS amendment were Rhizobiales and Eurotiales, respectively. These microorganisms promote plant growth by facilitating carbon and nitrogen utilization. In contrast, bacteria belonging to Saccharimonadales were the main responders at the maize silking and physiological maturity stages. Saccharimonadales facilitate soil organic matter cycling and the release of nutrients such as nitrogen and phosphorus. The nutrient requirements of plants differ according to the growth stage, leading to the recruitment of distinct key microorganisms. Our findings are consistent with previous work showing that responsive microbial communities exhibit nitrogen-fixing, cellulose-degrading, and lignin-degrading activities. Our results suggest that SCS is an excellent soil amendment that enhances soil nutrient levels and the establishment of beneficial microbial communities, leading to increased crop yields and the effective mitigation of soil degradation. Further investigation of the key beneficial microorganisms identified at the different growth stages individually or in combination with soil conditioning substances will provide a solid theoretical foundation for the targeted enhancement of crop yield and soil quality.

## Figures and Tables

**Table 1:** Soil chemical properties in the two treatments at the four maize growth stages.

Treatments	Growth Stage	pH	EC	TC (%)	TN (%)	C/N	TP (mg/kg)	AP (mg/kg)
CK	Seedling stage	6.45 ± 0.04 a	55.83 ± 1.21 b	2.02 ± 0.04 b	0.21 ± 0.01 b	9.5 ± 0.13 b	4.85 ± 0.18 b	2.14 ± 0.32 b
	Vegetative stage	6.49 ± 0.05 a	66.87 ± 4.41 b	3.22 ± 0.02 b	0.25 ± 0.02 b	12.78 ± 0.91 b	7.18 ± 0.06 b	3.16 ± 0.08 b
	Silking stage	5.96 ± 0.13 a	73.77 ± 0.42 b	2.5 ± 0.02 b	0.23 ± 0.02 b	10.94 ± 0.91 b	8.32 ± 0.45 a	3.48 ± 0.18 a
	Physiological maturity	6.33 ± 0.01 a	55.2 ± 2.01 b	1.71 ± 0.02 b	0.18 ± 0.02 b	9.35 ± 0.96 b	5.92 ± 0.32 b	2.75 ± 0.04 b
CM	Seedling stage	6.39 ± 0.08 a	169.83 ± 19.8 a	18.02 ± 0.02 a	0.79 ± 0.03 a	22.95 ± 0.83 a	10.34 ± 0.32 a	3.49 ± 0.17 a
	Vegetative stage	6.4 ± 0.02 b	549 ± 17 a	20.26 ± 0.02 a	0.93 ± 0.02 a	21.79 ± 0.53 a	16.7 ± 0.15 a	5.3 ± 0.42 a
	Silking stage	6.1 ± 0.02 a	85.37 ± 0.45 a	12.94 ± 0.01 a	0.59 ± 0.01 a	21.84 ± 0.19 a	6.78 ± 0.45 b	3.54 ± 0.18 a
	Physiological maturity	5.95 ± 0.13 b	124.6 ± 1.35 a	12.04 ± 0.04 a	0.57 ± 0.02 a	20.97 ± 0.6 a	10.55 ± 0.97 a	4.07 ± 0.01 a

TC, total carbon; TN, total nitrogen; TP, total phosphorus; AP, available phosphorus; pH, potential of hydrogen; EC, electrical conductivity; C/N, carbon/nitrogen ratio in organic matter. The results are given as the mean ± SD (standard deviation). Different lowercase letters within a column indicate significant differences at the *p* ≤0.05 level between the two treatments.

## Data Availability

The data used in this study are confidential.
